# *ADH1B* and *ADH1C* Genotype, Alcohol Consumption and Biomarkers of Liver Function: Findings from a Mendelian Randomization Study in 58,313 European Origin Danes

**DOI:** 10.1371/journal.pone.0114294

**Published:** 2014-12-15

**Authors:** Debbie A. Lawlor, Marianne Benn, Luisa Zuccolo, N. Maneka G. De Silva, Anne Tybjaerg-Hansen, George Davey Smith, Børge G. Nordestgaard

**Affiliations:** 1 MRC Integrative Epidemiology Unit at the University of Bristol, Bristol, United Kingdom; 2 School of Social and Community Medicine, University of Bristol, Bristol, United Kingdom; 3 The Copenhagen General Population Study, Herlev Hospital, Copenhagen, Denmark; 4 Copenhagen University Hospital, Faculty of Health and Medical Sciences, University of Copenhagen, Copenhagen, Denmark; 5 Department of Clinical Biochemistry, Herlev Hospital, Copenhagen, Denmark; 6 Department of Clinical Biochemistry, Rigshospitalet, Copenhagen, Denmark; Institute of Medical Research A Lanari-IDIM, University of Buenos Aires-National Council of Scientific and Technological Research (CONICET), Argentina

## Abstract

**Background:**

The effect of alcohol consumption on liver function is difficult to determine because of reporting bias and potential residual confounding. Our aim was to determine this effect using genetic variants to proxy for the unbiased effect of alcohol.

**Methods:**

We used variants in *ADH1B* and *ADH1C* genes as instrumental variables (IV) to estimate the causal effect of long-term alcohol consumption on alanine aminotransferase (ALT), γ-glutamyl-transferase (γ-GT), alkaline phosphatase (ALP), bilirubin and prothrombin action. Analyses were undertaken on 58,313 Danes (mean age 56).

**Results:**

In both confounder adjusted multivariable and genetic-IV analyses greater alcohol consumption, amongst those who drank any alcohol, was associated with higher ALT [mean difference per doubling of alcohol consumption: 3.4% (95% CI: 3.1, 3.7) from multivariable analyses and 3.7% (−4.5, 11.9) from genetic-IV analyses] and γ-GT [8.2% (7.8, 8.5) and 6.8% (−2.8, 16.5)]. The point estimates from the two methods were very similar and statistically the results from the two methods were consistent with each other for effects with ALT and γ-GT (both p_diff_>0.3). Results from the multivariable analyses suggested a weak inverse association of alcohol with ALP [−1.5% (−1.7, −1.3)], which differed from the strong positive effect found in genetic-IV analyses [11.6% (6.8, 16.4)] (p_diff_<0.0001). In both multivariable and genetic-IV analyses associations with bilirubin and protrombin action were weak and close to the null.

**Conclusions:**

Our results suggest that greater consumption of alcohol is related to poorer liver function as indicated by higher ALT, γ-GT and ALP, but not to clotting or bilirubin.

## Introduction

Alcoholic liver disease is a major global health problem, but the effects of lifetime differences in alcohol consumption in the general population are unclear [1]. It has been suggested that clinically apparent health damaging effects of consumption occur at levels of 80 g/day of alcohol for 10–12 years [2], but there is debate about whether lower levels also have adverse effects [3]. Most countries recommend safe drinking levels of up to 30 g/day for men and 20 g/day for women, but whether regular consumption at even these relatively low levels across a substantial part of adult life might result in liver damage is unclear [4].

Examining the association of long-term alcohol consumption with outcomes using conventional observational epidemiology methods is likely to be biased by: (i) confounding by characteristics related both to alcohol consumption and liver function/disease; (ii) ill health causing people to change their drinking behaviour (so called ‘reverse causality’) and (iii) systematic mis-reporting, such that those who are ill or are concerned about their drinking are likely to underreport intake [2]. Randomised controlled trials of different levels of long-term consumption of alcohol are not feasible. However, Mendelian randomization, in which genetic variants that are robustly related to alcohol intake are used as instrumental variables (IVs) to determine the life-time causal impact of different levels of intake, could help address this question [5], [6]. Results from this method are unlikely to be influenced by confounding or reverse causality [5], [7]. Simulation studies suggest that systematic under-reporting of an exposure used in a Mendelian randomization genetic-IV study can be biased away from the null [8]. However, since participants are extremely unlikely to know their genotype we expect the magnitude of this bias to be less than that of the multiple regression analysis, which would tend to bias results towards the null.

Alcohol is degraded to acetaldehyde in the liver by alcohol dehydrogenase and then subsequently to acetate by acetaldehyde dehydrogenase [9]. Changes in this degradation of alcohol that result in increased circulating and/or intracellular acetaldehyde levels cause unpleasant nausea and flushing in relation to alcohol consumption and as a consequence reduced levels of consumption [9]. In East Asian populations a common variant in the *ALDH2* gene, which encodes for acetaldehyde dehydrogenase 2, has been associated with alcohol consumption [10], [11]. Recent studies using this *ALDH2* genotype suggest that lifetime alcohol consumption, in a dose response fashion across the distribution, is related to higher ALT, AST and γ-GT in East Asian populations [12]–[14]. However, since this variant is not polymorphic in European populations it cannot be used to examine the effects of alcohol on liver function or disease endpoints in Europeans.

Genetic variants in the alcohol dehydrogenase (*ADH*) genes also produce between-person variation in the speed with which people degrade alcohol and its metabolites, and consequently influence alcohol reactions and levels of consumption [9], [15]. Amongst European origin populations, common functional polymorphisms in *ADH1B* and *ADH1C* are associated with levels of alcohol consumption; people who have fast degrading alleles consume less alcohol than those with slow degrading alleles [15]–[17]. Whilst there is some evidence that these variants are related to biomarkers of liver function and liver disease [18], [19], to our knowledge no study has used these variants as IVs to explore the causal effect of long-term alcohol consumption with liver outcomes.

The aim of this study was to use variants in *ADH1B* and *ADH1C* as genetic-IVs to estimate the causal effect of long-term alcohol consumption on biomarkers of liver function; alanine aminotransferase (ALT), γ-glutamyl-transferase (γ-GT), alkaline phosphatase (ALP), bilirubin and prothrombin action (assessed as the sum of action of coagulation factors II, VII and X, expressed as a percentage). These biomarkers/enzymes are used in clinical practice to indicate problems with liver function and the need for more invasive tests.

## Methods

We used data from the Copenhagen General Population Study, a large general population cohort study that aims to eventually recruit 100,000 participants and collect genotypic and phenotypic data of relevance to a wide range of health related problems. Individuals are randomly selected from the national Danish Civil Registration System and have to be aged 20 years or older and resident in greater Copenhagen; they also have to be white and of Danish decent. Recruitment began in 2003 and is still on-going. Additional study details have been previously published [6], [20], [21].

At the time of genotyping for the present study, 60,409 individuals had been recruited; 60,383 (99.9%) of these had adequate data on both genotypes and 58,313 (96%) had complete data on genotype, alcohol consumption and all potential confounding factors. These 58,313 provide our main analysis cohort. The study outcomes are all blood-based measurements and for most outcomes 99.9% of the 58,313 main analysis cohort had adequate measurements. For analyses with prothrombin action we removed the small number (1301/58,313, 2.2%) who had levels below 40% (indicating anticoagulation therapy) and so for this outcome analyses are completed on 57,012 participants.

Participants provided informed written consent and the study was approved by a Danish Medical Research Ethical Committee and by Herlev Hospital, Copenhagen University Hospital.

All measurements were completed by trained staff at one clinic centre. ALT, γ-GT, ALP, bilirubin and prothrombin action were measured using standard hospital assays (Konelab and ACL) and were subject to daily internal quality control assessing assay precision and monthly external quality control assessing assay accuracy. The laboratory staff completing these assays were blind to all participant characteristics, including age, gender, alcohol consumption, genotype or any known illnesses.

Amount of usual alcohol intake was reported as weekly consumption of beer in bottles and standard glasses of wine and spirits; each of these in Denmark contains the equivalent of ∼12 g of pure alcohol. Information on being a lifetime abstainer or whether those reporting no consumption had previously drunk but then stopped were not obtained and therefore those in the zero (no consumption) category are a mixture of these two. The *ADH1B* (rs1229984, Arg47His in exon 3) and *ADH1C* (rs698, Ile349Val in exon 8) genotypes were identified by TaqMan assays described in the supplementary web-material (**S1 File**). Genotype calls agreed with those using the Nanogen microelectronic chip technology [16], [22].

Details of how potential confounders were assessed have been reported in previous publications [6], [16], [20], [21] and in the supplementary web-material (**S1 File**).

### Statistical analyses

All analyses were conducted in Stata version 11. ALT, γ-GT, ALP and bilirubin were natural log-transformed to improve normality. An exact test was used to examine Hardy-Weinberg Equilibrium (HWE) and linkage disequilibrium between the two variants was assessed with Lewontin’s D' and r^2^ [23], [24].

Our *a priori* assumption was that the genetic variants would not be associated with potentially confounding factors of the alcohol-outcome associations, but we checked this using linear or logistic regression.

We used two approaches to examine the relationship of alcohol with outcomes - multivariable linear regression analyses and genetic-IV analyses, using *ADH1B* and *ADH1C* genotypes as instruments. Both methods produce the regression coefficients of liver function biomarkers on alcohol with the same units for both methods. Assuming the underlying assumptions of each method are correct, they both estimate the causal effect of alcohol consumption on each outcome. In multivariable analyses we examined associations across the whole distribution of alcohol consumption, including amongst those reporting no consumption, and tested for departure from linearity (see additional analysis methods in **S1 File**). Then in the main analyses we determined the graded dose response association of alcohol consumption with liver function in those consuming some alcohol and the mean difference in liver function between those who drank and those who reported not drinking any alcohol using both the multivariable and genetic-IV methods (the latter with *ADH1B* and *ADH1C* as IVs) and compared findings using these two methods. Full details of how these analyses were completed, including underlying assumptions are provided in the supplementary web-material (**S1 File**).

## Results

The distributions of all characteristics were the same in the 60,383 eligible participants and in the 58,313 participants with complete data who were included in the analyses (**S1 Table in S1 File**). Overall, 10% of the population reported drinking no alcohol and 9% reported drinking 25 or more drinks per week. Drinking differed by gender, with 13% of women reporting no consumption and 3% reporting 25 or more drinks per week, compared with 6% of men reporting no consumption and 17% reporting 25 or more drinks per week (p<0.0001).

Both genotypes were in HWE (p = 0.5 and 0.7 for *ADH1B* and *ADH1C*, respectively). The linkage disequilibrium coefficient Lewontin’s D' was 0.86 and r^2^ was 0.006 between *ADH1B* and *ADH1C*.

All of the observed confounders were associated with alcohol consumption (**S2 Table in S1 File**), but neither genotype was associated with any of the observed confounding factors (**Table 1**).

**Table 1:** Associations of observed confounders with *ADH1B* and *ADH1C* genotype.

	*ADH1B*			*ADH1C*			
	Mean (SD) or N (%) by genotype			Mean (SD) or N (%) by genotype			
	1/1 (slow)N = 55,880	1/2 or 2/2(fast) N = 2433	p-value^a^	2/2 (slow)N = 10,155	1/2 (intermediate)N = 28,415	1/1 (fast)N = 19,743	p-value^a^
Age (years)	56.6 (13.4)	57.1 (13.3)	0.13	56.6 (13.3)	56.7 (13.3)	56.6 (13.4)	0.55
Women (n, %)	31,144 (56)	1,324 (54)	0.20	5,741 (56)	15,818 (56)	10,909 (55)	0.11
Current smoker (n, %)	11,813 (21)	514 (21)	0.99	2,221 (22)	5,957 (21)	4,149 (21)	0.14
>4 hours per week MVPA	26,871 (48)	1,194 (49)	0.34	4,898 (48)	13,662(48)	9,505 (48)	0.96
Income >600,000Kr	10,767 (19)	499 (20)	0.13	1,946 (19)	5,535 (19)	3,785 (19)	0.64
Education >13 years	9,425(17)	416 (17)	0.76	1,776 (17)	4,738 (17)	3,327 (17)	0.17

**N = 58,313**.

a F-statistic for continuous variables and chi-square for categorical variables testing the null hypothesis that distributions of the confounders do not differ by genotype (1 degree of freedom for *ADH1B* and 2 degrees of freedom for *ADH1C*).

MVPA = Moderate or vigorous physical activity. Kr = Danish kroner.

### Multivariable associations in all participants

**Figure 1:**
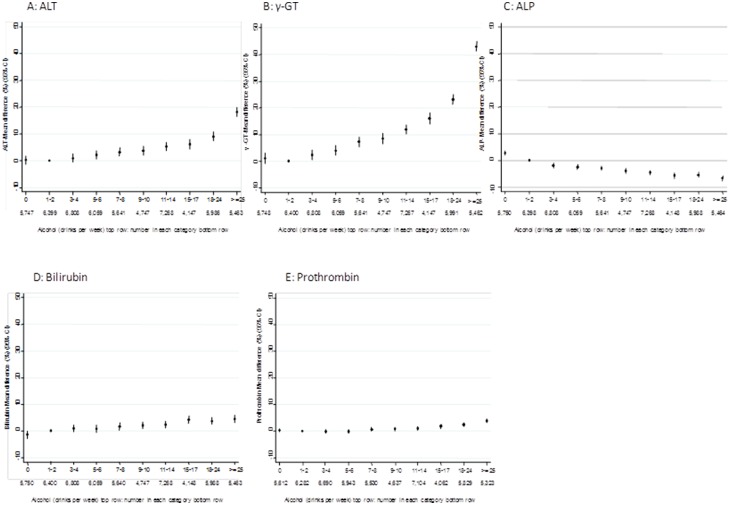
Multivariable associations of alcohol consumption with biomarkers of liver function. N = 58,313. All associations are adjusted for age, gender, physical activity, smoking, education and income. The reference category in all analyses is no drinks; this takes the null value of 0 for all outcomes.

**Fig. 1** shows the fully-adjusted associations of alcohol category with outcomes. Detailed age and gender (**S3 Table in S1 File**) and fully adjusted (**S4 Table in S1 File**) associations with all outcomes by alcohol category are shown in supplementary web material. In these analyses (that included all participants, including non-drinkers) alcohol consumption was positively associated with ALT, γ-GT, bilirubin and prothrombin action. There was also a weak inverse association with ALP (i.e. suggesting higher alcohol consumption is associated with lower ALP). Because of the large sample size for these observational analyses, p-values for linear trend and also for deviation from linearity are small. Amongst those drinking some alcohol, positive associations for ALT, γ-GT and inverse associations for ALP are monotonic. **Fig. 1** has the same scale for each outcome on the Y-axis and it can be seen from this, that despite all associations having very low p-values, the magnitude of the linear association in these multivariable analyses is weak/modest for all except ALT and γ-GT.

### Genetic-instrumental variable effects and their comparison with multivariable associations

**Figure 2:**
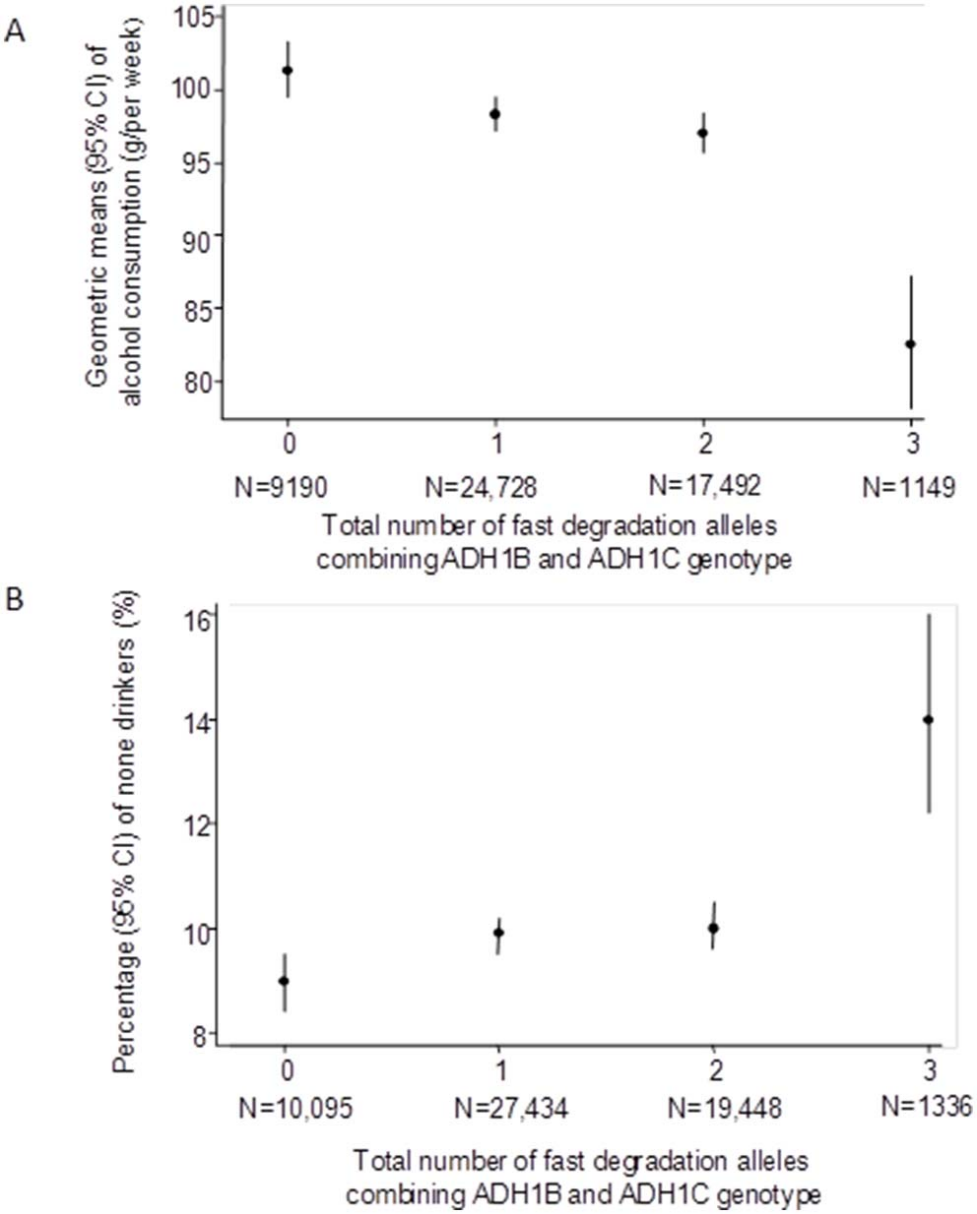
Association of combined *ADH1B* and *ADH1B* fast-allele score with alcohol consumption. N = 58,313. Shows geometric means (dots) and 95% confidence intervals of geometric means (vertical lines) of alcohol grams per week in those consuming some alcohol (A) and prevalence (%) (dots) and 95% confidence intervals of non-drinkers in the whole cohort (B) by total number of fast-alleles.

Individuals with greater numbers of fast degrading alleles were more likely to be non-drinkers and on average drank less alcohol if they reported some consumption (**Fig. 2** and **Table 2**). These associations are monotonic, but the difference between those with 3 or 4 alleles is greater than those between 0 and 1 or 1 and 2. Associations of *ADH1B*, *ADH1C* and the total allele score with alcohol consumption were similar in males and females (**S5a Table in S1 File**; p_interaction_ all ≥0.3). Genotypes appeared more strongly related to alcohol consumption at younger compared with older ages (**S5b Table in S1 File**), but there was no strong statistical evidence that associations differed by age (p_interaction_ all ≥0.1).

**Table 2:** Association of *ADH1B* and *ADH1C* with alcohol consumption.

	Mean difference (%) alcohol consumption inthose consuming some alcohol (95% CI) N = 52,559	OR of being a non-drinker in thewhole cohort (95% CI) N = 58,313
*ADH1B* one or two fast alleles versus none	–15.8 (–19.9, −11.8)	1.45 (1.28, 1.63)
R^2^	0.0011	
F-test	59	37^a^
P-value	<0.0001	<0.0001
*ADH1C* per fast allele	–2.2 (–3.3, −1.0)	1.06 (1.02, 1.10)
R^2^	0.0001	
F-test	14	9^a^
P-value	0.0002	0.003
Total *ADH1B* plus *ADH1C* allele score	–3.0 (–4.1, −1.9)	1.09 (1.05, 1.13)
R^2^	0.0012	
F-test	31	20^a^
P-value	<0.0001	<0.0001

OR: Odds ratio; CI: confidence intervals.

a From a model of the mean risk difference of not drinking.

The overidentification test results for all analyses provided evidence that the two SNPs are providing consistent IV results and are jointly valid instruments for alcohol consumption (p_overidentification_≥0.2). Associations using different IV analysis methods (**S6 Table in S1 File**) were similar to the main analyses using the control function.

In both confounder adjusted multivariable and genetic-IV analyses greater alcohol consumption, amongst those who drank any alcohol, was associated with higher ALT and γ-GT (**Table 3**). Whilst the sizes of the associations for the two analytical methods for these two outcomes were similar, with ALT increasing by 3–4% per doubling of alcohol intake and γ-GT by 7–8% per doubling of intake, the confidence intervals around the genetic-IV analysis results were very wide and included the null value. The weak (−1.5%) inverse association of alcohol with ALP in the multivariable analyses was in stark contrast to the positive association in the genetic-IV analyses (mean difference 11.6% (95% CI: 6.8, 16.4) per doubling of alcohol intake amongst those drinking some alcohol) and there was statistical evidence that these results differed from each other (p_difference_<0.001). In both multivariable and genetic-IV analyses associations with bilirubin and prothrombin action were weak and close to the null, being weakly positive in multivariable and null in genetic-IV analyses.

**Table 3:** Confounder adjusted multivariable and instrumental variable associations of alcohol with biomarkers of liver function in those who report some alcohol consumption (i.e. those reporting no consumption have been removed from these analyses).

	Mean difference in each outcome per doubling of alcohol (95% CI)				
	ALT (%)N = 52,518	γ-GT (%)N = 52,522	ALP (%)N = 52,521	Bilirubin (%)N = 52,521	Prothrombin (%)N = 51,400
Multivariable	3.4 (3,1, 3.7)	8.2 (7.8, 8.5)	–1.5 (–1.7, −1.3)	1.1 (0.8, 1.3)	0.8 (0.7, 0.9)
Instrumental variable	3.7 (–4.5, 11.9)	6.8 (–2.8, 16.5)	11.6 (6.8, 16.4)	–2.4 (–9.4, 4.7)	–1.8 (–5.3, 1.7)
P_difference_ instrumentalvariable vs. multivariable^a^	0.53	0.37	<0.0001	0.13	0.24

CI: confidence interval; ALT: alanine aminotransferase; γ-GT: γ-glutamyl-transferase; ALP: alkaline phosphatase; Prothrombin: Prothrombin action.

In the multivariable analysis all results are adjusted for age, gender, smoking, physical activity, education and income.

In the instrumental variable analysis the control function method was used with *ADH1B* and *ADH1C* used jointly as categorical (indicator) instrumental variables. The first stage F-statistic for all instrumental variable analyses = 34.

a Test of null hypothesis that there is no difference in association of alcohol with each outcome between the confounder adjusted multivariable association (row 1) and the instrumental variable association using the control function (row 2); p-value obtained from the bootstrap distribution.

**Table 4** shows the multivariable and genetic-IV analysis results comparing drinkers to non-drinkers. These results were broadly consistent with those examining associations of amount of consumption amongst drinkers, such that drinkers (of any amount) compared to non-drinkers had higher ALT and γ-GT in both multivariable and IV analyses, but with the latter being imprecisely estimated. There was a marked difference between the multivariable and IV analyses for ALP with the multivariable analyses suggesting a slightly lower level in drinkers but the genetic-IV analyses a marked higher level in drinkers compared to drinkers.

**Table 4:** Confounder adjusted multivariable and instrumental variable associations of drinking versus not drinking alcohol with biomarkers for liver function.

	Mean difference in each outcome comparing drinkers non-drinkers (95% CI)				
	ALT (%)N = 58,265	γ-GT (%)N = 58,270	ALP (%)N = 58,271	Bilirubin (%)N = 58,271	Prothrombin (%)N = 57,012
Multivariable	4.2 (2.9, 5.4)	9.5 (8.0, 11.0)	–6.1 (–6.9, −5.4)	3.4 (2.3, 4.4)	0.5 (0.0, 1.1)
Instrumental variable	28.1 (–19.0, 75.2)	57.8 (2.3, 113.3)	53.6 (26.1, 81.0)	0.4 (–39.5, 40.3)	–14.7 (–34.8, 5.4)
P_difference_ instrumentalvariable vs. multivariable^a^	0.44	0.11	<0.0001	0.61	0.22

CI: confidence interval; ALT: alanine aminotransferase; γ-GT: γ-glutamyl-transferase; ALP: alkaline phosphatase; Prothrombin: Prothrombin action.

In the multivariable analysis all results are adjusted for age, gender, smoking, physical activity, education and income.

In the instrumental variable analysis the control function method was used with *ADH1B* and *ADH1C* used jointly as categorical (indicator) instrumental variables. The first stage F-statistic for all instrumental variable analyses = 21.

a Test of null hypothesis that there is no difference in association of alcohol with each outcome between the confounder adjusted multivariable association (row 1) and the instrumental variable association using the control function (row 2); p-value obtained from the bootstrap distribution.

### Additional results

In the multivariable confounder adjusted analyses there was statistical evidence that associations differed between males and females for ALT, γ-GT and prothrombin action. For prothrombin action the point estimates were similar in males and females but because of the large sample size in this study it was possible to have small p-values even for small differences that would not be of clinical importance (**S7 Table in S1 File**). In the genetic-IV analyses there was no statistical evidence that associations differed by gender for any outcome and, for most, point estimates were very similar to each other (**S7 Table in S1 File**). However, the positive effect of alcohol on ALT was stronger in males compared to females in the genetic-IV analyses, as seen in the multivariable analyses (**S7 Table in S1 File**). There was no evidence that associations differed by age for either multivariable or genetic-IV analyses, and when analyses were repeated with the youngest age group (20–39 years) removed they were essentially the same as those presented in **Tables 3** **and** **4**.

The median (interquartile range) percentage contribution to total alcohol consumed was 69% (47, 92), 18% (0, 40) and 0% (0, 14) for wine, beer and spirits, respectively. The Spearman correlation coefficients for amount consumed between total alcohol and each of wine, beer and spirits were 0.83, 0.60 and 0.50, respectively (all p-values<0.00001). The pattern of association of *ADH1B* and *ADH1C* with wine and beer was similar to that with total alcohol consumption (**S1a and S1b Figures in S1 File**). Variants did not appear to be associated with amount of spirits consumed (**S1c Figure in S1 File**). Both the multivariable and IV analyses results were similar when either wine or beer were used instead of total alcohol (results available from authors on request). The multivariable analyses with amount of spirits consumed were similar to those with total alcohol (results available from authors), but since there was no association of variants with spirits we were not able to undertake IV analysis with this exposure.

Associations of *ADH1B* with observed confounders were similar amongst drinkers and non-drinkers, with the exception of smoking (**S8 Table in S1 File**). There was no strong statistical evidence of an interaction between *ADH1B* and alcohol consumption (none versus some) in their associations with any outcomes (**S8 Table in S1 File**).

## Discussion

In this large study we used genetic variants that are related to alcohol consumption as genetic-IVs to determine the effect of long-term alcohol intake on liver function. Our results show a monotonic association of greater alcohol consumption amongst those drinking some alcohol with higher ALT and γ-GT in multivariable analyses after controlling for observed confounders. The results from our genetic-IV analyses had very similar point estimates and were statistically consistent for these two outcomes. However, the confidence intervals of the genetic-IV estimates were wide and included the null value. Similar findings were obtained comparing drinkers (of any amount) to non-drinkers. Taken together these results suggest that incrementally greater long-term alcohol consumption (across its distribution) is causally related to poorer liver function as indicated by these two biomarkers.

For ALP, multivariable analyses suggested a weak inverse association whereas the genetic-IV analyses suggested a strong positive association (more alcohol consumption higher levels of ALP) and there was statistical evidence that results from these two methods differed from each other for this outcome. Elevated levels of ALP would be expected with hepatic or biliary dysfunction and so the positive association with alcohol seen in the genetic-IV analyses is more plausible than the inverse association in the multivariable analyses. ALP is not specific to the hepatobiliary system and circulating levels are also affected by bone turnover and a range of nutritional factors. Levels are reduced in the presence of low protein malnutrition and other nutrient deficiencies, including vitamin B_6_ and C, which are common with excessive alcohol consumption. Thus, it is possible that the weak inverse association in the multivariable analyses is due to confounding by lack of self-care, reflected in sedentary behaviour (and hence low bone turnover) and poor nutrition, which are associated with greater alcohol consumption and lower ALP. Such confounding is unlikely to bias the IV analysis results [5], [7]. As noted in the introduction, misclassification of alcohol consumption by participants who are ill or concerned about their alcohol consumption would tend to bias the multiple regression analyses towards the null and the genetic-IV analyses away from the null. However, it seems unlikely that these biases would produce a marked difference in ALP results, whereas for all of the other markers of liver function that we have assessed, results are consistent with each other for the two methods.

The stronger association of alcohol with ALT in men than women in both the multivariable and genetic-IV analyses may reflect real differences in their response to alcohol, but studies generally suggest women have more adverse effects to alcohol than do men [25], [26]. These differences could be chance findings and require further replication.

Recent studies in East Asian populations have used a Mendelian randomization approach, but with variants in those populations that have stronger associations with alcohol, and found strong evidence for dose-response effects of lifetime alcohol consumption on markers of liver function (ALT, AST and γ-GT) [12]–[14]. In two recent meta-analyses, including Asian, European, African and Native American populations, the variants that we have used as genetic-IVs in this study, were shown to have strong associations with a combined outcome of alcohol- dependency, abuse and poorer liver function [18], [19]. It is difficult to compare the findings from those studies with ours because of the combined outcome and the lack of a formal IV analyses testing the association of alcohol consumption with liver function. However, the associations were strongest in Asian populations and did not reach conventional levels of statistical significance in European populations, which may have reflected lack of statistical power given the considerably lower prevalence of the minor alleles in these populations.

We examined associations with different markers of liver function and where these are on the same scale have found some differences in the magnitude of effect. γ-GT is regarded as a useful biomarker for alcohol consumption and our results suggest alcohol has a stronger causal effect on γ-GT than ALT. The weak multivariable, and null genetic-IV, association of alcohol with bilirubin and prothrombin suggests that for these more severe markers of liver damage there might not be a continuous dose-response effect of alcohol, but possibly a threshold or multi-stage effect. Though, this would need further exploration.

We examined IV assumptions [5] and tried to reduce violation of these as much as possible in this study. Whilst alcohol consumption was associated with all of the measured confounders, the two genetic variants were not associated with any, suggesting the assumption that the genetic-IV is not related to confounders is plausible. Since this cohort consisted of white individuals of Danish descent, population stratification is unlikely, this is further supported by the lack of association of these variants with height (**Table 1**). It is conceivable that in social groups where there is pressure to consume alcohol any effect of the genetic variants will be over-ridden by this social pressure. However, we have shown that in the youngest age group (20–39 years), amongst whom this pressure might be greatest, the variants are if anything more strongly associated with consumption than at older ages. In our study the association of *ADH* fast alleles with alcohol, when both variants were simply summed together, appears to be largely driven by the difference in alcohol consumption between those with 3 or 4 alleles compared with all other individuals. However, our main results combined the two variants as separate categorical variables and these results were consistent with those from a weighted allele score IV (results in **S1 File**). Our results were also robust to different IV analysis methods. For all analyses the first-stage F-statistic did not suggest we had problems with weak instrument bias, though the genetic-IV analysis results were imprecisely estimated with wide confidence intervals. With the exception of ALT, discussed above, the results for all other markers of liver function were consistent with each other. Since the two approaches have differing sources of bias, this consistency further strengthens the likelihood that these results reflect the causal effect.

A key limitation is our inability to distinguish between lifetime abstainers and those who have stopped drinking because of ill-health amongst the non-drinkers. This would potentially bias the multivariable analyses more so than the genetic-IV analyses, but could result in some underestimation of true associations in both. Since the genetic variants can only affect amount of alcohol consumed amongst those who have tried drinking some alcohol, we would not expect them to be related to liver outcomes in those who have never consumed alcohol (see further discussion in **S1 File**) [27]. In our study we found no evidence of statistical interaction between *ADH1B* and alcohol consumption with outcomes (**S1 File**). This could be because the group who (at the time of recruitment) report that they do not drink alcohol includes many who had been heavy drinkers and stopped drinking because of alcohol related ill-health or it could be that we lack statistical power to detect an interaction between *ADH1B* and alcohol consumption. One study in Japanese men found that variants in the *ALDH2* genotype were more strongly associated with AST, ALT and γ-GT amongst heavy alcohol drinkers than in low level drinkers, with those reporting moderate levels of consumption having associations between these two [14]. However, the sample size in that study was small (N = 385) and we are not aware of any other studies that have explored the interactions we have examined in European populations.

A further limitation is our inability to examine associations with liver disease outcomes. This was because the prevalence of these in this cohort, based on hospital admissions or death certifications is low, with just 0.7% having evidence of any alcohol-related liver disease, and with most of these already being present at recruitment (only 0.2% had incident alcoholic liver disease or death). To our knowledge no other study has used a Mendelian randomization approach to examine long-term alcohol consumption with such hard outcomes. We have examined associations with a range of liver function markers that reflect different aspects of liver function but have not been able to include all markers of liver function. In particular AST has not been measured in this cohort. In general AST and ALT respond similarly to liver damage and disease, though AST may increase by a greater amount in response to alcohol excess.

To conclude, we have used genetic variants that are robustly associated with alcohol consumption as IVs to examine the causal effect of alcohol consumption on liver function. Our findings suggest that greater long-term alcohol consumption had a graded linear associated with more adverse liver enzyme levels in a dose response way. The magnitudes of the point estimate of cause effect from the Mendelian randomization analyses suggest quite a large effect. However, the confidence intervals are wide and often include the null value. This is because even with both variants used together, they explain only a small proportion of the variation in alcohol consumption and much larger Mendelian randomization studies would be required to obtain precise estimates from this method. We are not aware of any studies with such large samples sizes and relevant data. Of note for most of the markers assessed there is close similarity in the magnitude of associations between the confounder adjusted multivariable and the genetic-IV analyses, which given their different sources of bias, provides support for these estimates representing the magnitude of causal effect. These findings need further replication in larger samples.

## Supporting Information

S1 File contains all supplementary material.
(DOCX)
