# Supplementary material for: ADH1B and ADH1C Genotype, Alcohol Consumption and Biomarkers of Liver Function: Findings from a Mendelian Randomization Study in 58,313 European Origin Danes
Source: PLoS One. 2014 Dec 15;9(12):e114294. doi: 10.1371/journal.pone.0114294 (PMC4266606; doi:10.1371/journal.pone.0114294)
Supplement: S1 File — contains all supplementary material. (DOCX) [file pone.0114294.s001.docx]

**File S1: Supplementary material for**

*ADH1B* and *ADH1C* genotype, alcohol consumption and biomarkers of liver function: findings from a Mendelian Randomization study in 58,313 European origin Danes.

Debbie A Lawlor,^1,2^ Marianne Benn,^3,4,5^ Luisa Zuccolo,^1,2^ Maneka de Silva,^1,2^ Anne Tybjaerg-Hansen, ^3,5,6^ George Davey Smith,^1,2^ Børge G Nordestgaard. ^3,4,6^

^1^MRC Integrative Epidemiology Unit at the University of Bristol, UK

^2^ School of Social and Community Medicine, University of Bristol, UK

^3^The Copenhagen General Population Study, Herlev Hospital, Copenhagen, Denmark

^4^ Copenhagen University Hospital, Faculty of Health and Medical Sciences, University of Copenhagen, Denmark.

^5^Department of Clinical Biochemistry, Herlev Hospital, Copenhagen, Denmark

^6^Department of Clinical Biochemistry, Rigshospitalet, Copenhagen, Denmark

**Supplementary methods**

*Assessment of confounders*

Participants completed a questionnaire that recorded smoking and amounts smoked and ages of starting and quitting, which have been used to categorise participants as current, past and never smokers. Participants were also asked about leisure time physical activity (coded as < 2 hours moderate activity; 2-4 hours moderate activity; >4 hours moderate activity or 2-4 hours vigorous activity; >4 hours vigorous activity per week), annual income (coded as <100,000Kr; 100,000-399,000Kr; 400,000-599,000Kr; >=600,000Kr (100,000Kr ~ 17,000US$ / £10,500) and education (0-9; 10-12; >=13 year of completed education) in this questionnaire. Answers to the questionnaire were reviewed by the investigator with the participant at the day of the clinic assessment and any inconsistencies corrected or any participant queries clarified.

*Additional information on genotyping*

TaqMan Assays for allelic discrimination were ordered by design from Applied Biosystems(Foster City, California, USA) as “SNP genotyping assay mix” with the sequences as shown in Supplementary Web-table *genotyping* below. The reaction mix for PCR was prepared as suggested by the manufacturer using “TaqMan Universal PCR Master Mix, No AmpErase UNG”. PCR reaction was also performed as suggested by the manufacturer with a 10 minutes hold at 95°C, followed by 55 cycles of 15 seconds denaturing at 92°C and 1 minute annealing/extension at 60°C. Genotypes were read and scored using a 7600HT TaqMan system from Applied Biosystems.

***Supplementary statistical analyses methods***

***General details of multivariable and instrumental variable analyses***

Approximately 10% of participants reported no alcohol consumption and amongst those who reported some consumption this was markedly right skewed. We examined associations in three different ways: (a) a 10 level categorical variable with all non-drinkers in one category and drinkers split into 9 categories with approximately similar numbers in each; (b) a logged continuous variable of alcohol intake per week amongst those reporting some consumption and (c) a binary variable comparing drinkers to non-drinkers. Method (a) allowed us to examine the shape of the association (i.e. linear or non-linear) for all participants using multivariable analyses, but it was not possible to do this with instrumental variables analyses due to potential problems of weak instrument bias (the difference in consumption levels between genotype for some of these categories was very small). Method (b) allowed us to examine a dose response effect within those consuming some alcohol using both multivariable and instrumental variable analyses. In order to help with interpretation in these analyses we transformed the right skewed alcohol variable to log base 2 so that results are the change in outcome per doubling of alcohol consumption amongst drinkers. Method (c) allowed us to examine the association of not drinking alcohol versus any consumption using both analytical methods. All of the outcomes are presented as differences in means on a percentage scale; prothrombin action is measured as a percentage and all other variables were log-transformed (to ensure residuals in the model were approximately normal) and the resulting coefficients were back transformed to give a percentage difference per exposure. Having all outcomes on the same scale also allows comparison of the magnitudes of association between them.

In the multivariable analyses we used the category representing 1-2 drinks (12-24g) per week as the reference. This allows the nature of the relationship between amount of alcohol consumed and each outcome amongst those who drink any alcohol (i.e. the group in whom the main instrumental variable analyses) to be easily viewed.

In IV analyses we used the control function estimator and used *ADH1B* and *ADH1C* genotypes jointly as multiple instruments (as categorical variables). We used a Sargan type test of over-identification to check the joint validity of using the two variants together. This tests whether they give consistent estimates when used individually.

***Additional instrumental variable analyses***

In addition to using both variants jointly as two categorical variables, we also undertook analyses using the two genetic variants jointly as an allele score. We generated an allele score of total number of ‘fast’ alleles summing these across *ADH1B* and *ADH1C*. Because of small numbers of participants with four fast alleles (N=25) these were combined with those with three fast alleles for the purpose of showing the relationship between genetic variants and alcohol consumption (Figure 2 in the main paper).

In the instrumental variables analyses of the dose response association of alcohol consumption with risk factors, in addition to including the two genotypes as separate instrumental variables (main analyses) we also combined them into a single weighted allele score instrumental variable. Ideally, such weighted allele scores should be calculated using weights from external sources (i.e. the beta-coefficients for the association of these genotypes with alcohol consumption from a large independent study).(1) However, for these variants the largest publication to date that has included both variants is the same cohort that we have used here.(2) Other studies in European origin populations are all considerably smaller,(all fewer than 4,000 participants) and have only explored the association of *ADH1C* with alcohol consumption.(3-8) Therefore, we have used internal weights from our own study. In order to avoid over fitting we did this by splitting the cohort into two random samples each including 50% of the participants. We produce weights separately in each 50% random sample by regressing alcohol on each genotype. We then used the beta-coefficients from random sample 1 as weights for the genotypes in random sample 2, and vice versa. Within each sample after multiplying genotype allele number by the weight from the other random sample we then summed the weighted genotypes to produce a weighted allele score within each sample. We used this weighted allele score as an instrumental variable separately in the two random half sub-samples and then pooled the results using a fixed effect meta-analysis.

We also checked the control function instrumental variables approach by completing analyses using the two-stage least squares and generalised method of moments (using the ivregress command in Stata for both of these).

***Comparing multivariable and instrumental variable estimates***

To compare the results of the confounder adjusted multivariable (fully adjusted) association with that from the instrumental variable analysis, we used 1000 bootstrap replications[9] to estimate standard errors of the differences between the instrumental variable and the multivariable estimates that account for the correlation between the two sets of estimates. We calculated confidence intervals and p-values from these standard errors based on a normal approximation for the sampling distribution of the mean difference, after checking the bootstrap distributions were close to normal using normal probability plots.

*Assessing causality by exploring gene*alcohol interaction*

As additional secondary analysis we examined whether there was an interaction between alcohol (some versus none) and *ADH1B* in relation their associations with each outcome. This can potentially provide a further examination of causal associations, because *ADH1B* should only influence the amount of alcohol consumed in those who drink some alcohol. Since our assumption is that *ADH1B* is a valid instrument – i.e. it only affects outcome via its influence on how much someone drinks – for lifelong abstainers we would not expect a gene-outcome association. This is because amongst lifelong abstainers who have never tried alcohol the variant cannot influence the amount that they have drunk.(10)

These analyses were done as secondary analyses acknowledging that we have two potential problems. First, a problem that is referred to as ‘collider bias’ could occur.(11, 12) The name ‘collider bias’ comes from the use directed acyclic graphs (DAGs) to inform the analysis protocol and interpretation of results from which a ‘collision’ can be seen. Specifically, in this study ‘collider bias’ could occur because of our inability to distinguish lifelong abstainers from those who have quit drinking because of being hazardous drinkers in the past and stopping drinking because of the adverse effects of this level of drinking on their health. Such ‘sick-quitters’ are potentially more likely to have no fast alleles (i.e. since fast alleles are associated with drinking less alcohol those who drank heavily in the past are more likely to have been ‘genetically able’ to drink more than) and it is possible that in this group some outcomes will be worse because of their past drinking.(11) They may also have other adverse healthy behaviours that they have not quit; for example they may smoke heavily and have a poor diet. Second, even with our large sample size we may have low statistical power to detect a gene*alcohol interaction. In order to assess the first problem we examined the association of genotype with observe confounders within the two strata of none drinkers and drinkers (web-table 7 below); though this cannot provide information about inbalance with unobserved confounders. In order to increase statistical power we only examined this interaction with the *ADH1B* variant which has the strongest association with alcohol. Importantly, these analyses are secondary analyses and *a priori* we decided that whatever the results they should be treated with some caution.

**Table S1a: Distribution of participant characteristics (categorical variables) for all eligible participants (N=60,383) and those with complete data on all variables who are included in all other analyses (N =58,313)**

| **Characteristic** | | **n (%) in eligible participants**  **N = 60,383** | **n (%) in analysis participants**  **N = 58,313** |
| --- | --- | --- | --- |
| Gender | Women | 33,409 (55) | 32,468 (56) |
|  | Men | 26,974 (45) | 25,845 (44) |
|  | Missing^a^ | 0 | N/A |
| *ADH1B* | 1/1 (slow) | 57,860 (96) | 55,880 (96) |
|  | 1/2 and 2/2 (fast) | 2523 (4) | 2,433 (4) |
|  | Missing^a^ | 0 | N/A |
| *ADH1C* | 2/2 (slow) | 10,507 (17) | 10,155 (17) |
|  | 2/1 (intermediate) | 29,430 (49) | 28,415 (49) |
|  | 1/1 (fast) | 20,446 (34) | 19,743 (34) |
|  | Missing^a^ | 0 | N/A |
| Alcohol intake (drinks^b^/week) | 0 | 5,974 (10) | 5,754 (10) |
|  | 1-2 | 6,620 (11) | 6,407 (11) |
|  | 3-4 | 7,014 (12) | 6,810 (12) |
|  | 5-6 | 6,266 (10) | 6,062 (10) |
|  | 7-8 | 5,827 (10) | 5,647 (10) |
|  | 9-10 | 4,922 (8) | 4,749 (8) |
|  | 11-14 | 7,524 (12) | 7,271 (12) |
|  | 15-17 | 4,304 (7) | 4,150 (7) |
|  | 18-24 | 6,189 (10) | 5,997 (10) |
|  | >=25 | 5,665 (9) | 5,466 (9) |
|  | Missing^a^ | 78 (0.1) | N/A |
| Smoking | Never | 24,690 (41) | 23,665 (41) |
|  | Past | 22,735 (38) | 22,320 (38) |
|  | Current | 12,534 (21) | 12,327 (21) |
|  | Missing^a^ | 424 (0.7) | N/A |
| Leisure time physical activity hours per week of moderate (MA) or vigorous (VA) activity | < 2 MA | 4,709 (8) | 4,484 (8) |
|  | 2-4 MA | 26,655 (44) | 25,764 (44) |
|  | > 4 MA or 2-4 VA | 25,429 (42) | 24,622 (42) |
|  | > 4 hours VA | 3,590 (6) | 3,443 (6) |
|  | Missing^a^ | 0 | N/A |
| Education | <10 years | 17,566 (29) | 17,016 (29) |
|  | 10 to <13 years | 32,387 (54) | 31,456 (54) |
|  | ≥ 13 years | 10,160 (17) | 9,841 (17) |
|  | Missing^a^ | 270 (0.4) | N/A |
| Income | <100,000 Kr | 1,151(2) | 1,110 (2) |
|  | 100,000-399,999Kr | 23,210 (38) | 22,799 (39) |
|  | 400,000-599,999Kr | 23,579 (39) | 23,138 (40) |
|  | ≥ 600,000Kr | 11,544 (19) | 11,266 (19) |
|  | Missing^a^ | 899 (2) | N/A |

^a^ For all categories the % missing is from the total eligible (N=60,383); the % in each non-missing category is from those with data on that variable (i.e. 58,313 minus number missing is the denominator). Kr= Danish kroner. ^b^ 1 drink is equivalent of ~12g of pure alcohol. NA= not applicable; this column is the complete case sample and so there is no missing data.

**Table S1b: Distribution of participant characteristics (continuously measured variables) for all eligible participants (N= 60,383) and those with complete data on all variables who are included in all other analyses (N = 58,313)**

| **Characteristic** | **Number with data (n, % missing out of the 60,383 eligible participants)** | **Mean (SD) or Median (IQR) in eligible participants**  **N = 60,383** | **Number with data (n, % missing out of the 58,313 analysis participants)** | **Mean (SD) or Median (IQR) in analysis participants**  **N = 58,313** |
| --- | --- | --- | --- | --- |
| Age (years) | 60,383 (0, 0) | 57 (13) | 58313 (0,0) | 57 (13) |
| ALT (U/L)^a^ | 60,332 (51, 0.1) | 20 (15, 28) | 58,265 (48, 0.1) | 20 (15, 28) |
| γ-GT (U/L)^a^ | 60,341 (42, 0.1) | 28 (21, 42) | 58272 (41, 0.1) | 28 (21, 42) |
| ALP (U/L)^a^ | 60,339 (44, 0.1) | 81 (68, 97) | 58271(42, 0.1) | 81 (68, 97) |
| Bilirubin (µmol/L)^a^ | 60338 (45,0,1) | 11 (8, 14) | 58271 (42, 0.1) | 11 (8, 14) |
| Prothrombin activity^b^ | 59021 (1362, 2) | 102 (20) | 57012  (1301, 2.2) | 102 (20) |
| Alcohol (grams/week) amongst drinkers^a,c^ | 54,331 | 108 (48, 192) | 52559 | 108 (48, 192) |

^a^ Distributions for these variables are median (interquartile range; IQR) because the distributions are right skewed; all other results are means (standard deviation; SD)

^b^ Prothrombin activity is the precentage combined activity of factors II, VII and X

^c^ This is the distribution only for those reporting consumption of some alcohol - since this is restricted only to those with some consumption missing data is not applicable here (Web-table 1a shows extent of missing alcohol consumption data when all levels (including non-drinking) are considered).

.

**Table S2: Association of observed confounders with alcohol consumption. N = 58,313**

|  | **Mean (SD) or N (%) by categories of alcohol consumption (drinks**^b^ **per week)** | | | | | | | | | | **p-value^a^** |
| --- | --- | --- | --- | --- | --- | --- | --- | --- | --- | --- | --- |
|  | **0**  **N = 5,754** | **1-2**  **N = 6,407** | **3-4**  **N = 6,810** | **5-6**  **N = 6,062** | **7-8**  **N = 5,647** | **9-10**  **N = 4,749** | **11-14**  **N = 7,271** | **15-17**  **N = 4,150** | **18-24**  **N = 5,997** | **>= 25**  **N = 5,466** |  |
| Age (years) | 55.8 (15.1) | 53.1(14.5) | 53.6 (13.9) | 54.8 (13.6) | 56.6 (13.4) | 57.1 (12.8) | 58.1 (12.5) | 59.1 (11.9) | 59.8 (11.7) | 59.9 (10.9) | < 0.0001 |
| Women (n, %) | 4,227 (73) | 4,771 (74) | 4,584 (67) | 3,807 (63) | 3,413 (60) | 2,708 (57) | 3,744 (51) | 1,949  (47) | 2,166 (36) | 1,099 (20) | < 0.0001 |
| Current smoker  (n, %) | 1,639 (28) | 1,277 (20) | 1,238 (18) | 1,042 (17) | 945 (17) | 877 (18) | 1,299 (18) | 861 (21) | 1,389 (23) | 1,760 (32) | < 0.0001 |
| Income > 600,000Kr | 468 (8) | 973 (15) | 1,378 (20) | 1,247 (21) | 1,218 (22) | 1,044 (22) | 1,645 (23) | 926 (22) | 1,332 (22) | 1,035 (19) | < 0.0001 |
| Education > 13 years | 904 (16) | 1,286 (20) | 1,365 (20) | 1,164 (19) | 968 (17) | 789  (16) | 1,163  (16) | 636 (15) | 870 (14) | 696 (13) | < 0.0001 |
| >4 hours per week MVPA | 2,119 (37) | 2,728 (43) | 3,239 (48) | 2,935 (48) | 2,896 (51) | 2,375 (50) | 3,781 (52) | 2,128 (51) | 3,157 (53) | 2,707 (49) | < 0.0001 |

^a^ F-statistic for continuous variables and chi^2^ for categorical variables testing the null hypothesis that distributions of the confounders do not differ by alcohol category (9 degrees of freedom)

^b^ 1 drink is equivalent of ~12g of pure alcohol.

MVPA: Moderate or vigorous physical activity

**Table S3: Age and gender adjusted association of alcohol with biomarkers of liver function.**

| **Alcohol drinks/week (median grams)** | **Mean difference of each outcome by alcohol category (95% CI)** | | | | |
| --- | --- | --- | --- | --- | --- |
|  | **ALT (%)**  **N= 58,265** | **γ-GT (%)**  **N= 58,270** | **ALP (%)**  **N= 58,271** | **Bilirubin (%)**  **N= 58,271** | **Prothrombin (%)**  **N= 57,012** |
| 0 (0) | 0.2 (-1.5, 1.8) | 3.2 (1.3, 5.1) | 4.3 (3.3, 5.3) | -3.4 (-4.8, -2.0) | 1.6 (0.9, 2.3) |
| 1-2 (18g) | Ref = 0 | Ref = 0 | Ref = 0 | Ref = 0 | Ref = 0 |
| 3-4 (42g) | 0.7 (-0.9, 2.2) | 1.2 (-0.6, 3.0) | -2.8 (-3.7, -1.9) | 1.7 (0.3, 3.0) | -0.8 (-1.5, -0.1) |
| 5-6 (66g) | 2.0 (0.4, 3.6) | 2.5 (0.6, 4.4) | -3.5 (-4.5, -2.6) | 1.7 (0.4, 3.1) | -0.9 (-1.7, -0.3) |
| 7-8 (90g) | 2.8 (1.2, 4.5) | 5.2 (3.3, 7.1) | -4.3 (-5.3, -3.3) | 2.9 (1.5, 4.3) | -0.5 (-1.2, 0.2) |
| 9-10 (114g) | 3.2 (1.5, 4.9) | 6.4 (4.4, 8.4) | -5.2 (-6.3, -4.2) | 3.1 (1.6, 4.5) | -0.3 (-1.0, 0.4) |
| 11-14 (150g) | 4.7 (3.1, 6.2) | 9.4 (7.6, 11.2) | -6.2 (-7.1, -5.3) | 3.5 (2.2, 4.8) | -0.3 (-1.0, 0.3) |
| 15-17 (192g) | 5.2 (3.4, 7.0) | 13.7 (11.6, 15.8) | -7.2 (-8.2, -6.1) | 5.1 (3.6, 6.6) | 0.5 (-0.3, 1.2) |
| 18-24 (252g) | 7.8 (6.2, 9.5) | 20.6 (18.7, 22.5) | -6.9 (-7.9, -5.9) | 4.4 (3.0, 5.8) | 1.1 (0.4, 1.8) |
| ≥ 25 (324g) | 16.5 (14.8, 18.2) | 41.9 (39.9, 43.9) | -7.1 (-8.2, -6.1) | 3.6 (2.1, 5.1) | 3.4 (2.7, 4.1) |
| Mean difference per category | 1.4 (1.2, 1.5) | 3.4 (3.2, 3.5) | -1.1 (-1.2, -1.0) | 0.7 (0.6, 0.8) | 0.2 (0.1, 0.2) |
| p-linear ^a^ | <0.0001 | <0.0001 | <0.0001 | <0.0001 | <0.0001 |
| p-non linear^b^ | <0.0001 | <0.0001 | <0.0001 | <0.0001 | <0.0001 |

ALT: alanine aminotransferase; γ-GT: γ-glutamyl-transferase; ALP: alkaline phosphatase; Prothrombin: Prothrombin action.

^a^ F-statistic for null hypothesis that there is no linear trend across alcohol categories; categories fitted as a score (1 degree of freedom).

^b^ Likelihood ratio test for null hypothesis of deviation from linearity across alcohol categories (8 degrees of freedom).

**Table S4: Fully adjusted association of alcohol with biomarkers of liver function.**

| **Alcohol drinks/week (median grams)** | **Mean difference of each outcome by alcohol category (95% CI)** | | | | |
| --- | --- | --- | --- | --- | --- |
|  | **ALT (%)**  **N=58,265** | **γ-GT (%)**  **N=58,270** | **ALP (%)**  **N= 58,271** | **Bilirubin (%)**  **N= 58,271** | **Prothrombin (%)**  **N=57,012** |
| 0 (0) | 0.3 (-1.3, 1.9) | 1.1 (-0.8, 3.0) | 2.8 (1.8, 3.7) | -1.4 (-2.8, -0.1) | 0.3 (-0.3, 1.0) |
| 1-2 (18g) | Ref = 0 | Ref = 0 | Ref = 0 | Ref = 0 | Ref = 0 |
| 3-4 (42g) | 0.9 (-0.6, 2.5) | 2.4 (0.6, 4.2) | -2.0 (-2.9, -1.1) | 0.9 (-0.3, 2.3) | -0.1 (-0.8, 0.5) |
| 5-6 (66g) | 2.2 (0.6, 3.8) | 3.9 (2.1, 5.8) | -2.6 (-3.5, -1.6) | 0.8 (-0.5, 2.2) | -0.2 (-0.9, 0.5) |
| 7-8 (90g) | 3.3 (1.6, 4.9) | 7.3 (5.4, 9.2) | -3.0 (-3.9, -2.0) | 1.7 (0.3, 3.1) | 0.6 (-0.1, 1.3) |
| 9-10 (114g) | 3.7 (2.0, 5.4) | 8.4 (6.4, 10.4) | -4.0 (-5.0, -2.9) | 2.1 (0.7, 3.5) | 0.8 (0.1, 1.5) |
| 11-14 (150g) | 5.3 (3.7, 6.8) | 11.9 (10.1, 13.7) | -4.6 (-5.5, -3.7) | 2.3 (1.0, 3.6) | 1.0 (0.4, 1.7) |
| 15-17 (192g) | 6.1 (4.3, 7.9) | 16.0 (14.0, 18.1) | -5.7 (-6.8, -4.6) | 4.2 (2.7, 5.7) | 1.8 (1.0, 2.6) |
| 18-24 (252g) | 9.0 (7.4, 10.7) | 23.2 (21.3, 25.1) | -5.5 (-6 .4, -4.5) | 3.7 (2.4, 5.1) | 2.4 (1.7, 3.1) |
| ≥ 25 (324g) | 18.1 (16.4, 19.8) | 43.0 (41.1, 45.0) | -6.7 (-7.7, -5.7) | 4.5 (3.1, 6.0) | 3.9 (3.1, 4.6) |
| Mean difference per category | 1.5 (1.4, 1.7) | 3.8 (3.6, 3.9) | -0.9 (-1.0, -0.8) | 0.6 (0.5, 0.7) | 0.4 (0.3, 0.4) |
| p-linear ^a^ | <0.0001 | <0.0001 | <0.0001 | <0.0001 | <0.0001 |
| p-non linear^b^ | <0.0001 | <0.0001 | <0.0001 | 0.53 | <0.0001 |

ALT: alanine aminotransferase; γ-GT: γ-glutamyl-transferase; ALP: alkaline phosphatase; Prothrombin: Prothrombin action.

^a^ F-statistic for null hypothesis that there is no linear trend across alcohol categories; categories fitted as a score (1 degree of freedom).

^b^ Likelihood ratio test for null hypothesis of deviation from linearity across alcohol categories (8 degrees of freedom).

All results are adjusted for gender, age, smoking, income, education and physical activity.

**Table S5a: Association of *ADH1B* and *ADH1C* with alcohol consumption in males and females**

|  | **Mean difference (%) alcohol consumption in those consuming some alcohol (95%CI)** | | **OR of being a drinker in the whole cohort (95%CI)** | |
| --- | --- | --- | --- | --- |
|  | Females  N = 28,241 | Males  N = 24,318 | Females  N = 32,468 | Males  N =25,845 |
| *ADH1B* one or two fast alleles versus none | -16.6 (-22.1, -11.2) | -18.1 (-23.5,-12.6) | 0.64 (0.55, 0.74) | 0.80 (0.63, 1.01) |
| *ADH1C* per fast allele | -2.0 (-3.5, -0.5) | -3.3 (-4.9, -1.7) | 0.93 (0.88, 0.97) | 0.96 (0.89, 1.03) |
| Total *ADH1B* plus *ADH1C* allele score | -2.9 (-.4.3, -1.5) | -4.2 (-5.7, -2.7) | 0.90 (0.86, 0.94) | 0.95 (0.88, 1.01) |

OR: Odds ratio; CI: confidence intervals.

**Table S5b: Association of *ADH1B* and *ADH1C* with alcohol consumption in different age groups**

|  | **Mean difference (%) alcohol consumption in those consuming some alcohol (95%CI)** | | | **OR of being a drinker in the whole cohort (95%CI)** | | |
| --- | --- | --- | --- | --- | --- | --- |
|  | Age 20-39 years  N = 4211 | Age 40-59 years  N =25,833 | Age ≥ 60  N =22,515 | Age 20-39 years  N =5013 | Age 40-59 years  N =28,386 | Age ≥ 60  N =24,914 |
| *ADH1B* one or two fast alleles versus none | -19.7 (-34.8, -4.6) | -21.9 (-27.6, -16.2) | -10.6 (-16.5, -4.7) | 0.58 (0.41, 0.82) | 0.66 (0.55, 0.78) | 0.75 (0.62, 0.90) |
| *ADH1C* per fast allele | -5.3 (-9.2, -1.5) | -2.5 (-4.1, -0.9) | -1.1 (-2.8, 0.6) | 0.90 (0.81, 1.01) | 0.95 (0.89, 1.01) | 0.95 (0.89, 1.01) |
| Total *ADH1B* plus *ADH1C* allele score | -5.9 ( -9.6, -2.3) | -3.7 (-5.2, 2.2) | -1.7 (-3.3, -0.1) | 0.89 (0.79, 0.97) | 0.93 (0.88, 0.97) | 0.93 (0.88, 0.99) |

OR: Odds ratio; CI: confidence intervals.

**Table S6: Instrumental variable sensitivity analyses - associations of alcohol with biomarkers of liver function in those who report some alcohol consumption (i.e. those reporting no consumption have been removed from these analyses), using *ADH1B* and *ADH1C* together as an internally weighted allele score and using different instrumental variable methods.**

|  | **Mean differences (95%CI) of outcomes per doubling of alcohol consumption in those reporting some alcohol consumption** | | | | |
| --- | --- | --- | --- | --- | --- |
|  | **ALT (%)**  **N=52,518** | **γ-GT (%)**  **N=52,522** | **ALP (%)**  **N=52,521** | **Bilirubin (%)**  **N=52,521** | **Prothrombin (%)**  **N=51,400** |
| *ADH1B* and *ADH1C* jointly as categorical variables^a, b^ | 3.7  (-4.5, 11.9) | 6.8  (-2.8, 16.5) | 11.6  (6.8, 16.4) | -2.4  (-9.4, 4.7) | -1.8  (-5.3, 1.7) |
| *ADH1B* and *ADH1C* jointly as weighted allele score^a^ | 3.3  (-5.1, 11.7) | 5.9  (-4.0, 15.8) | 12.3  (7.4, 17.1) | -3.2  (-10.4, 3.9) | -1.0  (-4.6, 2.6) |
| *ADH1B* and *ADH1C* jointly as categorical variables using 2SLS | 3.7  (-4.6, 12.2) | 6.8  (-3.2, 16.8) | 11.7  (6.0, 17.4) | -2.4  (-9.7, 4.9) | -1.7  (-5.3, 1.8) |
| *ADH1B* and *ADH1C* jointly as categorical variables using LIML | 3.7  (**-**4.7, 12.2) | 6.8  (-3.2, 16.8) | 11.7  (6.0, 17.5) | -2.4  (-9.8, 4.9) | -1.9  (-5.5, 1.8) |

ALT: alanine aminotransferase; γ-GT: γ-glutamyl-transferase; ALP: alkaline phosphatase; Prothrombin: Prothrombin action.

^a^ The control function method for the instrumental variables analyses was used in all of these analyses.

2SLS: 2-stage least squares method; LIML: limited information maximum likelihood method.

^b^ This is the model used in the main paper.

First-stage F test for both variants used as separate IVs (i.e. main analyses in top row and in 2SLS and LIML) = 34; First-stage F test for weighted allele score = 38 and 27 in the two random 50% samples, respectively (see methods for deriving this score above)

**Table S7: Confounder adjusted multivariable and instrumental variable associations of alcohol with biomarkers of liver function in those who report some alcohol consumption (i.e. those reporting no consumption have been removed from these analyses), stratified by sex.**

|  | **Mean difference in each outcome per doubling of alcohol (95% CI)**  **The null value for all results =0** | | | | |
| --- | --- | --- | --- | --- | --- |
|  | **ALT (%)** | **γ-GT (%)** | **ALP (%)** | **Bilirubin (%)** | **Prothrombin action (%)** |
| Multivariable (female) | 2.3 (1.9, 2.7) | 5.7 (5.2, 6.2) | -1.5 (-1.8, -1.3) | 1.2 (0.9, 1,6) | 0.6 (0.5, 0.8) |
| Multivariable (male) | 4.8 (4.3, 5.3) | 11.1 (10.6, 11.7) | -1.4 (-1.7, -1.1) | 0.8 (0.4, 1.2) | 1.1 (0.9, 1.3) |
| P_interaction with sex_^a^ | <0.0001 | <0.0001 | 0.55 | 0.15 | 0.001 |
| Instrumental variable (female) | 1.6  (-9.0, 12.3) | 9.7  (-2.9, 22.2) | 10.8  (3.0, 18.6) | 4.8  (-5.0, 14.5) | 0.0  (-4.8, 4.9, 0.9) |
| Instrumental variable (male) | 11.6  (1.4, 21.9) | 10.7  (-1.2, 22.6) | 10.8  (4.4, 17.1) | -3.3  (-11.8, 5.3) | -3.6  (-8.0, 0.8) |
| p_iv(female)_ vs. p_iv(male)_^b^ | 0.23 | 0.91 | 0.99 | 0.33 | 0.35 |

CI: confidence interval; ALT: alanine aminotransferase; γ-GT: γ-glutamyl-transferase; ALP: alkaline phosphatase; Prothrombin: Prothrombin action.

In the multivariable analysis all results are adjusted for age, gender, smoking, physical activity, education and income.

In the instrumental variable analysis the limited information maximum likelihood method (LIML) was used with *ADH1B* and *ADH1C* used jointly as categorical (indicator) instrumental variables.

^a^ Test of null hypothesis that there is no difference in association of alcohol with each outcome in the confounder adjusted multivariable association between females (row 1) and males (row 2); obtained from entering an interaction term in the multivariable model.

^b^ Test of null hypothesis that there is no difference in association of alcohol with each outcome in the instrumental variable analyses between in females (row 4) and males (row 5); p-value obtained from the bootstrap distribution.

**Table S8: Association of *ADH1B* fast alleles with observed confounding factors in those reporting no alcohol consumption and those reporting some alcohol consumption**. **N=58,313**

| **Outcome** | **Odds ratio (95%CI) comparing one or two to no fast alleles in non-drinkers**  **N = 5754** | **Odds ratio (95%CI) comparing one or two to no fast alleles**  **in drinkers**  **N = 52559** | **p-value_interaction_^a^** |
| --- | --- | --- | --- |
| Female | 0.89 (0.68, 1.15) | 1.11 (1.02, 1.22) | 0.10 |
| Current smoker | 0.79 (0.61, 1.02) | 1.02 (0.92, 1.14) | 0.07 |
| High physical activity | 1.03 (0.82, 1.30) | 1.06 (0.98, 1.16) | 0.80 |
| High income | 1.42 (0.99, 2.04) | 1.09 (0.98, 1.22) | 0.17 |
| High education | 0.84 (0.61, 1.17) | 1.05 (0.93, 1.17) | 0.22 |
| Age (years)^b^ | -0.07 (-1.76, 1.61) | 0.54 (-0.03, 1.11) | 0.45 |

Note: the associations are of the fast degrading alleles, which are associated with lower alcohol consumption and therefore odds ratios greater than one suggest greater odds in those who drink **less** alcohol and odds ratios less than one suggest lower odds in those who drink **more** alcohol

^a^ p-value_interaction_ testing the null hypothesis that the association of *ADH1B* with each observed confounder is the same in those reporting that they drink no alcohol at the time of questionnaire administration as in those who report some alcohol consumption

^b^ Mean difference in years (95%CI) comparing one or two to no fast alleles.**Table S9: Association of *ADH1B* fast alleles with biomarkers of liver function in those reporting no alcohol consumption and those reporting some alcohol consumption**.

| **Outcome** | **Mean difference (95%CI) comparing two to no fast alleles**  **in non-drinkers** | **Mean difference (95%CI) comparing two to no fast alleles**  **in drinkers** | **p-value_interaction_^a^** |
| --- | --- | --- | --- |
| **ALT (%)** | 2.1 (-3.4, 7.7) | -0.8 (-2.9, 1.2) | 0.30 |
| **γ-GT (%)** | -0.4 (-6.3, 5.5) | -1.5 (-3.9, 1.0) | 0.76 |
| **ALP (%)** | 1.0 (-2.2, 4.2) | -2.7 (-3.9, -1.5) | 0.03 |
| **Bilirubin (%)** | -1.0 (-5.5, 3.6) | 0.8 (-0.9, 2.6) | 0.46 |
| **Prothrombin action (%)** | 1.9 (-0.4, 4.2) | 0.7 (-0.1, 1.6) | 0.33 |

Note: the associations are of the fast degrading alleles with each outcome and therefore positive associations suggest higher mean outcome levels in those who drink less alcohol, and negative associations suggest higher mean outcome levels in those who drink more alcohol

^a^ p-value_interaction_ testing the null hypothesis that the association of *ADH1B* with each outcome is the same in those reporting that they drink no alcohol at the time of questionnaire administration as in those who report some alcohol consumption

**Supplementary material references:**

1. Pierce BL, Ahsan H, VanderWeele TJ. Power and instrument strength requirements for Mendelian randomization studies using multiple genetic variants. *International Journal of Epidemiology* 2011;40:740-752.
2. Tolstrup JS, Nordestgaard BG, Rasmussen S, Tybjaerg-Hansen A, Gronbaek M. Alcoholism and alcohol drinking habits predicted from alcohol dehydrogenase genes. Pharmacogenomics J 2008;8:220-27.
3. Hines LM, Stampfer MJ, Ma J, Gaziano JM, Ridker PM, Hankinson SE, Sacks F, Rimm EB, Hunter DJ. Genetic variation in alcohol dehydrogenase and the beneficial effect of moderate alcohol consumption on myocardial infarction. *N Engl J Med* 2001;344:549–55
4. Whitfield JB, O’Brien ME, Nightingale BN, Zhu G, Heath AC, Martin NG. ADH genotype does not modify the effects of alcohol on high density lipoprotein. *Alcohol Clin Exp Res* 2003;27:509–14
5. Djousse L, Levy D, Herbert AG, Wilson PW, D’Agostino RB, Cupples LA, Karamohamed S, Ellison RC. Influence of alcohol dehydrogenase 1C polymorphism on the alcohol-cardiovascular disease association (from the Framingham Offspring Study). *Am J Cardiol* 2005;96:227–32
6. Younis J, Cooper JA, Miller GJ, Humphries SE, Talmud PJ. Genetic variation in alcohol dehydrogenase 1C and the beneficial effect of alcohol intake on coronary heart disease risk in the Second Northwick Park Heart Study. *Atherosclerosis* 2005;180:225–32
7. Ebrahim S, Lawlor DA, Ben-Shlomo Y, Timpson N, Harbord R, Christensen M, Baban J, Keissling M, Day INM, Gaunt T, Davey Smith G. Alcohol dehydrogenase type 1C (ADL2C) variants, alcohol consumption, HDL-cholesterol and risk of coronary heart disease in women and men: British Women’s Heart and Health Study and Caerphilly cohorts. Atherosclerosis 2008;196:227-32
8. Latella MC, Di Castelnuovo A, de Lorgeril M, Arnout J, Cappucio FP, van Doungen MB, de Gaetano G, Lacoviella L, European Collaborative Group of the IMMIDIET Project. Genetic variation of alcohol dehydrogenase type 1C (ADH1C), alcohol consumption, and metabolic cardiovascular risk factors: Results from the IMMIDIET study. *Atherosclerosis* 2009;207:284-90.
9. Efron B, Tibshirani RJ. An introduction to the bootstrap. New York: Chapman & Hall; 1993
10. Davey Smith G. Use of genetic markers and gene-diet interactions for interrogating population-level causal influences of diet on health. *Genes and Nutrition* 2011;6:27-43
11. Glymour MM, Tchetgen Tchetgen EJ, Robins JM. Credible Mendelian randomization studies: approaches for evaluating the instrumental variables assumptions. *American Journal of Epidemiology* 2012;175:332-339
12. Cole SR, Platt RW, Schisterman EF, Chu H, Westreich D, Richardson D, Poole C. Illustrating bias due to conditioning on a collider. International Journal of Epidemiology 2010; 39:417-420.
